# Negative Regulation of the Keap1-Nrf2 Pathway by a p62/Sqstm1 Splicing Variant

**DOI:** 10.1128/MCB.00642-17

**Published:** 2018-03-15

**Authors:** Shun Kageyama, Tetsuya Saito, Miki Obata, Ryo-hei Koide, Yoshinobu Ichimura, Masaaki Komatsu

**Affiliations:** a Department of Biochemistry, Niigata University Graduate School of Medical and Dental Sciences, Niigata, Japan

**Keywords:** autophagy, Keap1, Nrf2, RNA splicing, p62/Sqstm1

## Abstract

A key antioxidant pathway, the Keap1-Nrf2 system, is regulated by p62/Sqstm1 via multiple mechanisms, including gene expression, posttranslational modifications (such as ubiquitination and phosphorylation), and autophagic degradation of p62/Sqstm1 and Keap1. Here we demonstrate a novel mode of regulation of the Keap1-Nrf2 system, mediated by a splicing variant of p62/Sqstm1 pre-mRNA. Ensembl database searches and subsequent biochemical analyses of mice revealed the presence of an mRNA that encodes a p62/Sqstm1 protein lacking the Keap1-interacting region (KIR), which is essential for the interaction with Keap1. Like full-length p62, the variant was induced under conditions in which Nrf2 was activated (e.g., impairment of autophagy), formed oligomers with itself and/or the full-length protein, and was degraded by autophagy. However, the variant failed to interact with Keap1 and sequester it in variant-positive aggregates. Remarkably, while full-length p62 stabilized Nrf2 and induced the gene expression of Nrf2 targets, the variant increased the amount of Keap1 and enhanced ubiquitination of Nrf2, thereby suppressing the induction of Nrf2 targets. Hepatocytes isolated from genetically modified mice that express full-length p62, but not the variant, were susceptible to activation of Nrf2 in response to stress. Collectively, our results suggest that splicing of p62/Sqstm1 pre-mRNA negatively regulates the Keap1-Nrf2 pathway.

## INTRODUCTION

The Keap1-Nrf2 pathway is a major oxidative stress response system (1, 2). Nuclear factor erythroid 2-related factor 2 (Nrf2) is a basic leucine zipper transcription factor, and Kelch-like ECH-associated protein 1 (Keap1) is an adaptor protein of cullin-3-based ubiquitin ligase. In the canonical Keap1-Nrf2 pathway, Keap1 acts as a sensor and Nrf2 serves as an effector. Under normal conditions, a Keap1 homodimer recognizes the ETGE and DLGex motifs of an Nrf2 molecule via the same binding pocket, located on the bottom surface of Keap1 (3–6). Subsequently, Nrf2 is ubiquitinated and rapidly degraded by the 26S proteasome. Upon exposure to oxidative stress and electrophilic insults, specific cysteine residues of Keap1 are modified by oxidants, and the Keap1 homodimer loses its two-site binding affinity for Nrf2 (7–10). As a result, Nrf2 escapes from the Keap1 interaction and translocates into the nucleus, where it induces a battery of genes encoding antioxidant proteins and detoxifying enzymes.

Several noncanonical pathways for Nrf2 activation involve competitive inhibition of the Keap1-Nrf2 interaction by intracellular proteins, including p62/Sqstm1 (referred to here as p62) (11), PGAM5 (12), and CIP/WAF1 (13). Among them, one characterized pathway is the p62-Keap1-Nrf2 axis. p62, a stress-inducible cellular protein, has multiple domains that mediate its interactions with various binding partners, and it serves as a signaling hub for diverse cellular events, such as amino acid sensing and the oxidative stress response (14–16). In addition, p62 functions as a selective autophagy receptor for degradation of ubiquitinated substrates (17, 18). In the p62-Keap1-Nrf2 axis, p62 functions as a modulator of Nrf2 activation. The Keap1-interacting region (KIR) of p62 binds to Keap1 in a manner similar to that for the ETGE motif of Nrf2, thereby preventing Keap1 from trapping Nrf2, resulting in Nrf2 stabilization and activation (11, 19). This noncanonical pathway is strictly modulated at multiple steps, including gene expression (20), posttranslational modification (21, 22), and turnover (23, 24). The *p62* gene is a target of Nrf2 (20), implying that a positive-feedback loop exists within the p62-Keap1-Nrf2 axis. Serine 349, located at the KIR of p62 (corresponding to mouse serine 351), is phosphorylated under stress conditions, and phosphorylated p62 has a higher affinity for Keap1 (21), indicating that phosphorylation of p62 is sufficient for disruption of Keap1-mediated Nrf2 ubiquitination. Autophagy is directly involved in this pathway: p62 is degraded by autophagy in a p62 aggregation-dependent manner (25), and Keap1 is also degraded by autophagy in a p62 interaction- and aggregation-dependent fashion (23). Lysine 7 of the N-terminal Phox1 and Bem1p (PB1) domain of p62 is ubiquitinated by TRIM21, which prevents p62 aggregation and subsequent Keap1 sequestration on the aggregate structures (22). Thus, TRIM21 negatively regulates the p62-Keap1-Nrf2 axis.

Here we describe negative regulation of the p62-Keap1-Nrf2 axis by a p62 variant that lacks the KIR. We identified a *p62* splicing variant in which exon 7 is partially skipped, and this variant encodes a KIR-deleted p62 protein. The splicing product neither interacted with Keap1 nor sequestered Keap1 on variant-positive aggregates. In marked contrast to that of full-length p62, overproduction of the variant suppressed Nrf2 activity. Hepatocytes in which the variant was deleted were more susceptible to induction of Nrf2. Taken together, our results suggest that splicing of *p62* pre-mRNA negatively regulates the p62-Keap1-Nrf2 axis.

## RESULTS

### *p62* splicing variant lacking the Keap1-interacting region.

**FIG 1:**
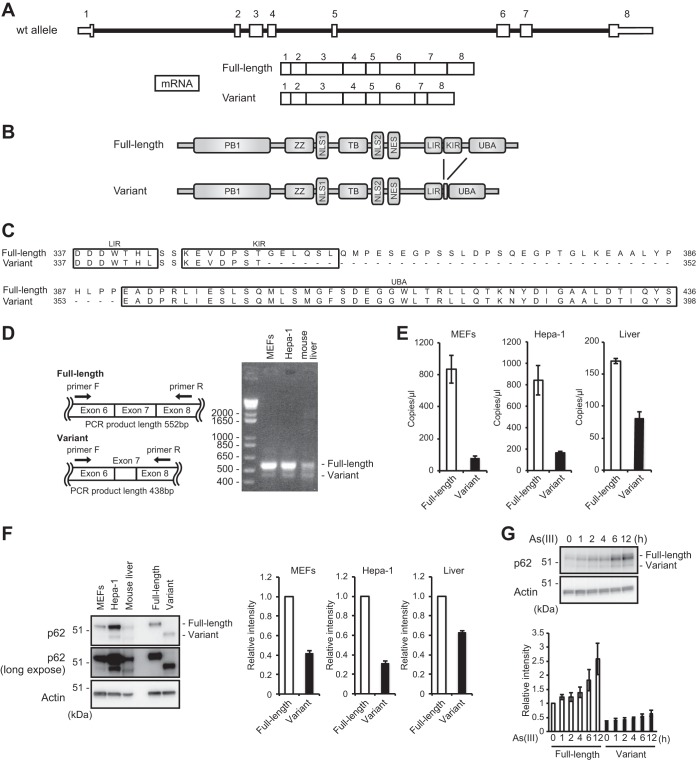
A p62 splicing variant lacking the Keap1-interacting region is present in mice. (A) Schematic diagram of genome structures of mouse *p62*. Coding exons, numbered in accordance with the initiation site (exon 1), are depicted by white boxes. The full-length mRNA and its splicing variant produced from the *p62* allele are shown. wt, wild type. (B) Domain structures of mouse full-length and variant p62 proteins. Phox and Bem1 (PB1) mediates homo-oligomerization or hetero-oligomerization. The LC3-interacting region (LIR) and the C-terminal ubiquitin-associated (UBA) domain promote the sequestration of ubiquitinated substrates into the autophagosome. The Keap1-interacting region (KIR) binds to Keap1. ZZ, zinc finger; TB, TRAF6-binding domain; NLS, nuclear localization signal; NES, nuclear export signal. (C) Alignment of regions containing the LIR, the KIR, and the UBA domain in the full-length and variant p62 proteins of mice. (D) RT-PCR. Analyses of *p62* exon 7 splicing in MEFs, Hepa-1 cells, and mouse liver were performed. Diagrams at left indicate cDNAs encoding full-length and variant p62 proteins. The positions of primers used for RT-PCR analysis of *p62* cDNA and the expected sizes of the PCR products in the presence and absence of the last half of exon 7 are shown. The right panel shows the results of gel electrophoresis (2% agarose gel) of RT-PCR products from cDNAs of MEFs, Hepa-1 cells, and mouse liver. (E) Digital PCR analysis. RNA copy numbers for full-length p62 and its variant in MEFs, Hepa-1 cells, and mouse liver were determined using a QuantStudio 3D digital PCR system. (F) Immunoblot analysis. Total cell lysates of MEFs and Hepa-1 cells and mouse liver homogenate were prepared and subjected to immunoblot analysis with anti-p62 antibody. Nontagged full-length or variant p62 was expressed in *p62*-deficient HeLa cells, and the lysates were used as positive controls. Data are representative of three independent experiments. Quantitative densitometry analysis of immunoblotting data was performed, and the levels of full-length p62 and its variant were normalized against that of actin. (G) Immunoblot analysis. Primary mouse hepatocytes were challenged with 10 μM sodium arsenite [As(III)] for the indicated times. Data are representative of three independent experiments. Quantitative densitometry analysis of immunoblotting data was performed, and the levels of full-length p62 and its variant were normalized against that of actin.

The mouse Ensembl database (http://www.ensembl.org/Mus_musculus/Info/Index) predicted the presence of a variant of *p62* mRNA in which the last half of exon 7 of the full-length *p62* mRNA is skipped. This prediction was made at transcript support level 1, indicating that all splice junctions of the transcript are supported by at least one nonsuspect mRNA (Fig. 1A). Interestingly, the variant encodes a p62 protein that lacks the last half of the KIR and the linker region between the KIR and the ubiquitin-associated (UBA) domain (Fig. 1B and C). We confirmed the presence of not only full-length *p62* but also the variant mRNA in mouse embryonic fibroblasts (MEFs), a mouse hepatocellular carcinoma cell line (Hepa-1), and mouse liver by reverse transcriptase PCR (RT-PCR) (Fig. 1D). Digital PCR analysis also supported the presence of the variant mRNA (Fig. 1E). In comparison with full-length p62 mRNA, MEFs and Hepa-1 cells hardly expressed the variant. In contrast, in mouse liver, the RNA copy number of the variant was relatively large, i.e., approximately half that of the full-length isoform (Fig. 1E). Immunoblot analysis with anti-p62 antibody confirmed the existence of both variant p62 and full-length p62 (Fig. 1F). Quantitation of the data revealed that the level of the variant form relative to that of the full-length protein was higher in mouse liver than in MEFs and Hepa-1 cells (Fig. 1F). Considering that the two forms share a promoter and that *p62* is an Nrf2 target, we speculated that as with full-length p62, expression of the variant is induced by exposure to oxidative stressors, such as arsenite, known to be a potent activator of Nrf2. In fact, both forms accumulated in arsenite-treated cells, in a time-dependent manner (Fig. 1G). Our results indicate the presence of a variant of p62 lacking the KIR and suggest that the gene expression mechanism is identical to that of the full-length protein.

### Oligomerization of the p62 variant with full-length p62.

**FIG 2:**
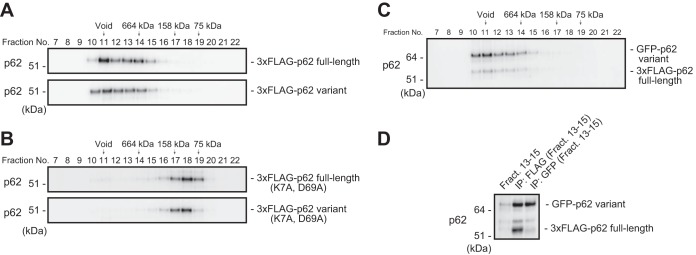
The p62 splicing variant forms a complex with full-length p62. (A) Gel filtration chromatography. FLAG-tagged p62 or its variant was expressed in *p62*-knockout MEFs. Lysates were subjected to gel filtration chromatography followed by immunoblot analysis with anti-p62 antibody. Data are representative of three independent experiments. (B) Gel filtration chromatography. FLAG-tagged PB1 mutant p62 or its variant form was expressed in *p62*-knockout MEFs. Lysates were subjected to gel filtration chromatography followed by immunoblot analysis with anti-p62 antibody. Data are representative of three independent experiments. (C) Gel filtration chromatography. FLAG-tagged full-length p62 and GFP-tagged variant p62 were coexpressed in *p62^−/−^* MEFs, and cell lysates were subjected to gel filtration chromatography followed by immunoblot assay with anti-p62 antibody. Data are representative of three independent experiments. (D) Immunoprecipitation (IP) assay. Fractions 13 to 15 from the gel filtration chromatography shown in panel B were subjected to immunoprecipitation with anti-FLAG or anti-GFP antibody. The immunoprecipitates were examined by immunoblotting with anti-p62 antibody. Data are representative of three independent experiments.

Next, we examined whether the p62 variant has the ability to form a self-oligomer or a hetero-oligomer with full-length p62. Oligomerization is indispensable not only for autophagic degradation of p62 (25, 26) but also for full activation of Nrf2 through p62 (11, 26). When we expressed full-length or variant p62 in *p62*-deficient MEFs, gel filtration chromatography revealed that both forms appeared in a high-molecular-mass fraction (above 660 kDa) (Fig. 2A). In both cases, the large protein complex became smaller (∼100 kDa) upon mutation of PB1 (K7A, D69A) (Fig. 2B). We further investigated whether the high-molecular-mass complex contained both full-length and variant p62 proteins. To this end, we expressed FLAG-tagged full-length p62 along with the green fluorescent protein (GFP)-tagged variant in *p62^−/−^* MEFs and then performed gel filtration chromatography. Similar to the results for single expression (Fig. 2A), we detected the majority of both forms in fractions above 660 kDa (Fig. 2C). Immunoprecipitation assay of fractions 13 to 15 revealed that both forms interacted with each other (Fig. 2D). These results suggest that these two forms interact and form a large complex in a PB1-dependent manner.

### The p62 variant does not interact with Keap1.

**FIG 3:**
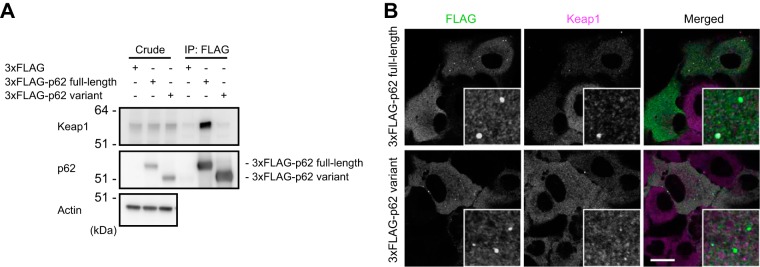
The p62 variant lacks the ability to bind Keap1. (A) Immunoprecipitation assay. FLAG-tagged full-length or variant p62 was expressed in *p62^−/−^* MEFs, and cell lysates were subjected to immunoprecipitation with anti-FLAG antibody. The immunoprecipitates were examined by immunoblotting with the indicated antibodies. Data are representative of three independent experiments. (B) Immunofluorescence analysis. 3×FLAG-tagged p62 or its variant was expressed in *p62*-knockout Huh1 cells by use of an adenovirus vector system, and the cells were immunostained with anti-FLAG and anti-Keap1 antibodies. Each inset is a magnified image. Bar, 20 μm.

The direct interaction of p62 with Keap1 is essential for Nrf2 activation (11, 19). Because the variant lacks the last half of the KIR, including glycine 353 (G353) and glutamate 354 (E354) (Fig. 1C), both of which form hydrogen bonds with Keap1 (11, 21), the variation was predicted to abolish the interaction with Keap1. Indeed, while FLAG-tagged full-length p62 interacted with endogenous Keap1, the FLAG-tagged variant did not (Fig. 3A). To further examine the dynamics of the variant in cells, we carried out immunofluorescence analysis (Fig. 3B). When the 3×FLAG-tagged full-length or variant protein was expressed in *p62*-knockout Huh1 cells, FLAG-positive aggregates with diameters ranging from 0.5 to 1 μm appeared in the cytosol (Fig. 3B). In agreement with biochemical analyses (Fig. 3A), Keap1 colocalized with the aggregates positive for full-length p62 but not with those positive for the variant (Fig. 3B).

### The p62 variant is degraded by autophagy.

**FIG 4:**
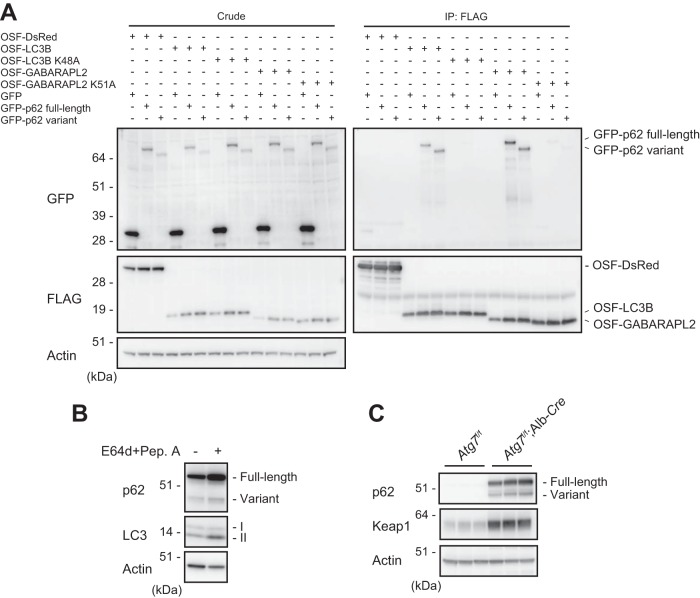
The p62 variant is degraded by autophagy. (A) Immunoprecipitation assay. One-Strep-FLAG (OSF)-tagged LC3B, GABARAPL2, and the corresponding hydrophobic pocket mutants were coexpressed with GFP-tagged full-length or variant p62 in HEK293T cells. Precipitates generated with anti-FLAG antibody were subjected to immunoblot analysis with anti-GFP and anti-FLAG antibodies. Data are representative of three independent experiments. (B) Immunoblot analysis. Primary hepatocytes were cultured in the presence or absence of E64d and pepstatin A (Pep. A) for 24 h. The lysates were subjected to NuPAGE followed by immunoblot analysis with the indicated antibodies. Data are representative of three independent experiments. (C) Immunoblot analysis. Homogenates prepared from livers of control *Atg7^f/f^* and *Atg7^f/f^*; albumin-*Cre* mice were analyzed by immunoblotting with the indicated antibodies. Data are representative of three independent experiments.

Full-length p62 in complex with Keap1 is degraded primarily by autophagy, in an LC3 interaction-dependent manner (23, 25, 27). The LC3-interacting region (LIR) of p62 is located just N-terminal to the KIR (25, 27) (Fig. 1B), and the lack of the KIR might affect the ability to bind ATG8 family proteins (LC3s and GABARAPs) that localize on the autophagosome (28, 29). To test this idea, we coexpressed One-Strep-FLAG-tagged LC3B or GABARAPL2 with full-length or variant p62. As shown in Fig. 4A, both forms of p62 bound to LC3B and GABARAPL2. However, these interactions were barely detectable in cells expressing LC3B (K48A) and GABARAPL2 (K51A) mutants which do not form the hydrophobic pocket required for the LIR interaction (25). These results suggest that the variant interacts with LC3B and GABARAPL2 in an LIR-dependent manner and is degraded by autophagy. Consistent with this result, treatment of MEFs with the lysosomal inhibitors E64d and pepstatin A caused significant accumulation of not only full-length p62 but also the variant form (Fig. 4B). Furthermore, both forms dramatically accumulated in livers of *Atg7^f/f^*; albumin-*Cre* mice, in which autophagy-related gene 7 (*Atg7*) is specifically deleted in hepatocytes (30) (Fig. 4C). We also observed a prominent accumulation of Keap1 in autophagy-deficient livers (Fig. 4C).

### Suppression of Nrf2 activity by the p62 variant.

**FIG 5:**
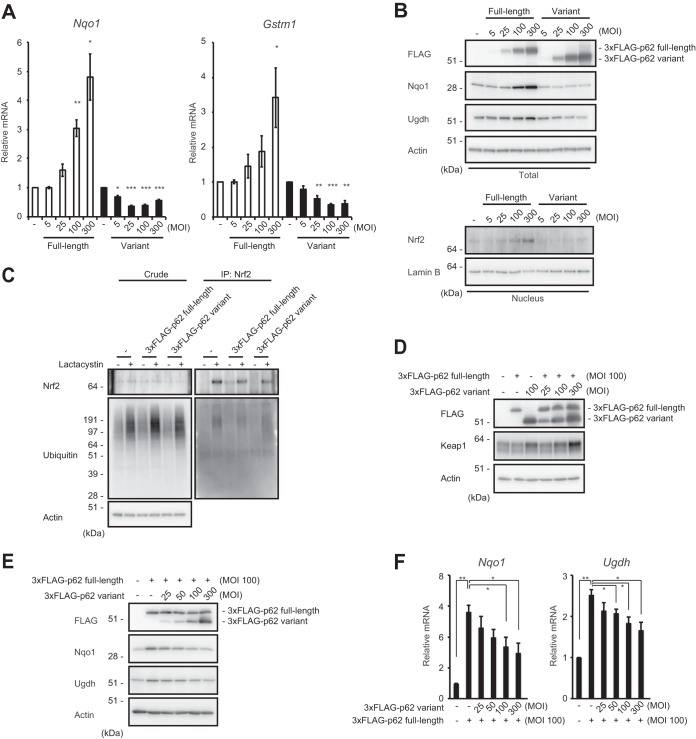
The p62 variant increases the amount of Keap1 and represses Nrf2 activity. (A) Real-time PCR. Relative mRNA levels of Nrf2 targets in primary mouse hepatocytes expressing full-length p62 or variant p62 at the indicated multiplicities of infection (MOIs) are shown. Values were normalized to the amount of each mRNA in the nontreated wild-type hepatocytes. The experiments were performed three times. Data are means ± standard errors of the means (SEM). *, *P* < 0.05; **, *P* < 0.01; ***, *P* < 0.001 (as determined by Welch's *t* test). (B) Immunoblot analysis. Cytosolic and nuclear fractions were prepared from the hepatocytes prepared as described for panel A and subjected to immunoblot analysis with the indicated antibodies. Data are representative of three independent experiments. (C) Ubiquitination assay with Nrf2. FLAG-tagged p62 and its variant were overproduced in primary mouse hepatocytes. Cells were cultured in the absence or presence of 10 μM lactacystin for 4 h. The cell lysates were immunoprecipitated with anti-Nrf2 antibody, subjected to electrophoresis in a NuPAGE gel, and analyzed by immunoblotting with anti-Nrf2 and antiubiquitin antibodies. Data are derived from three separate experiments. (D) Immunoblot analysis. Total lysates were prepared from primary mouse hepatocytes expressing full-length p62 or its variant, alone or in combination, at the indicated MOIs and then subjected to immunoblot analysis. Data are representative of three independent experiments. (E) Immunoblot analysis. Total lysates were prepared from *p62*-deficient primary mouse hepatocytes expressing full-length p62 alone or together with the variant at the indicated MOIs. The lysates were subjected to immunoblot analysis. Data are representative of three independent experiments. (F) Real-time PCR. Relative mRNA levels of Nrf2 targets in hepatocytes prepared as described for panel E are shown. Values were normalized to the amount of mRNA in nontreated *p62*-deficient hepatocytes. The experiments were performed three times. Data are means ± SEM. *, *P* < 0.05; **, *P* < 0.01 (as determined by Welch's *t* test).

In the next series of experiments, we examined the effect of the p62 variant on Nrf2 activation. As previously reported (11), adenovirus vector-mediated p62 expression in mouse primary culture hepatocytes resulted in the induction of Nrf2 target genes, such as the NAD(P)H dehydrogenase quinone 1 gene (*Nqo1*) and the glutathione *S*-transferase m1 gene (*Gstm1*) (Fig. 5A). In marked contrast, such induction was not observed when the variant was introduced. Instead, the variant suppressed the expression of *Nqo1* and *Gstm1*, in a dose-dependent fashion (Fig. 5A). We also confirmed the upregulation of Nrf2 target gene products, such as Nqo1 and UDP-glucose dehydrogenase (Ugdh), upon introduction of full-length p62 and downregulation of the same proteins upon introduction of the variant form (Fig. 5B). As expected, expression of full-length p62 increased the amount of nuclear Nrf2, in a dose-dependent manner, but this effect was barely detectable when the variant was overexpressed (Fig. 5B). We then tested the E3 activity of cullin-3-based Keap1 ubiquitin ligase against Nrf2 in the presence or absence of the p62 variant. The level of ubiquitination of Nrf2 was significantly higher in hepatocytes harboring the variant than in control hepatocytes or hepatocytes expressing full-length p62 (Fig. 5C). Keap1 is a substrate for autophagy, and the interaction of Keap1 with p62 is indispensable for its autophagic degradation (21, 23), raising the possibility that expression of a variant lacking the ability to interact with Keap1 impairs this degradation. To test this hypothesis, we expressed full-length p62 or the variant, alone or in combination, in wild-type primary cultured hepatocytes and examined the level of Keap1. As shown in Fig. 5D, the amount of Keap1 increased upon expression of the variant. This effect was observed even in cells overexpressing full-length p62, and it depended on the amount of the variant expressed (Fig. 5D). These results imply that the E3 activity of Keap1 is increased by expression of the variant.

Next, we investigated the extent to which the variant protein is needed to attenuate the effect of full-length p62 on Nrf2 activation. To this end, full-length p62 was expressed at a constant level, alone or along with the variant at a range of levels (Fig. 5E). The expression of Nrf2 targets induced by expression of full-length p62 in *p62*-deficient hepatocytes was suppressed by concomitant expression of the variant, in a concentration-dependent manner (Fig. 5F). The variant exhibited the inhibitory effect when it was present at half the level of the full-length protein (Fig. 5F). Consistent with this result, the levels of Nqo1 and Ugdh, both of which were significantly upregulated by expression of full-length p62, decreased depending on the expression level of the variant (Fig. 5E).

### Physiological significance of the p62 variant in mouse hepatocytes.

**FIG 6:**
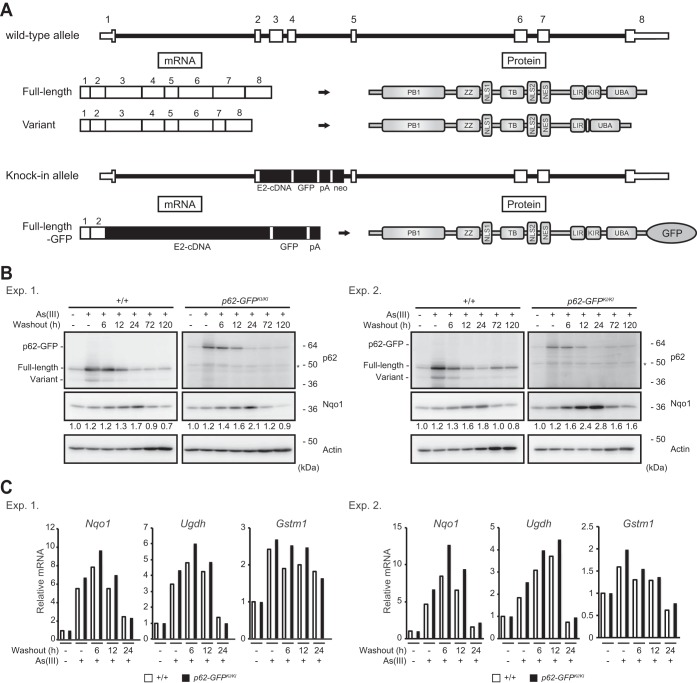
The p62 variant negatively regulates the p62-Keap1-Nrf2 axis in mouse hepatocytes. (A) Schematic diagram of the genome structures of wild-type *p62* and *p62-GFP* knock-in alleles. Coding exons, numbered in accordance with the initiation site (exon 1), are depicted by white boxes. E2-cDNA–GFP–pA indicates the *p62* cDNA fragment (positions 302 to 1326) fused with *GFP* cDNA and simian virus 40 (SV40) poly(A). mRNAs and proteins produced from each allele are shown. (B) Immunoblot analysis. Primary hepatocytes prepared from both wild-type and *p62-GFP* knock-in mice were challenged with 10 μM sodium arsenite [As(III)] for 12 h. After removal of As(III), cells were cultured in regular medium for the indicated times. Cell lysates were prepared and subjected to immunoblot analysis with the indicated antibodies. Data show the results of two independent experiments. Numerical values indicate the results of quantitative densitometric analyses of Nqo1 normalized against the levels in nontreated cells. An asterisk indicates a nonspecific band. (C) Real-time PCR. Relative mRNA levels of Nrf2 targets in hepatocytes prepared as described for panel B are shown. Values were normalized against the corresponding mRNA levels in nontreated wild-type or *p62-GFP* knock-in hepatocytes. The experiments were performed two times.

To elucidate the biological relevance of the variant, we used *p62-GFP* knock-in mice (*p62-GFP^KI/KI^*), in which the *p62* splicing variant cannot be produced (31) (Fig. 6A). Like wild-type hepatocytes, primary hepatocytes isolated from *p62-GFP^KI/KI^* mice expressed high levels of full-length p62-GFP in response to treatment with arsenite (Fig. 6B). Although the variant form was clearly detected in arsenite-treated wild-type hepatocytes, it was not observed in *p62-GFP^KI/KI^* hepatocytes (Fig. 6B). After removal of arsenite, the amounts of both full-length p62 and the variant in wild-type hepatocytes decreased in a time-dependent manner (Fig. 6B). Likewise, removal of arsenite caused a decrease in full-length p62-GFP in the knock-in hepatocytes (Fig. 6B). Next, we examined the effect of loss of the p62 variant on expression of Nrf2 target genes. In wild-type hepatocytes, expression of Nrf2 targets, such as *Nqo1*, *Ugdh*, and *Gstm1*, was induced by exposure to arsenite, reaching a maximum 6 h after removal of arsenite and diminishing gradually thereafter (Fig. 6C). *p62-GFP^KI/KI^* hepatocytes exhibited a similar expression pattern, but the induction of Nrf2 target genes 0, 6, and 12 h after treatment with arsenite was usually greater than that in wild-type hepatocytes (Fig. 6C). Consistent with the results of gene expression analysis, the level of the Nqo1 protein after removal of arsenite was higher in *p62-GFP^KI/KI^* hepatocytes than in wild-type hepatocytes (Fig. 6B). Taken together, these findings indicate that the p62 variant serves as a negative regulator of the p62-Keap1-Nrf2 axis under physiological conditions.

## DISCUSSION

In this study, we discovered a novel mode of regulation of the Keap1-Nrf2 pathway, mediated by splicing of *p62* pre-mRNA to generate a variant form lacking the last half of the KIR, in mice. Similar to full-length p62, the variant can self-oligomerize and interact with LC3B and GABARAPL2, and both forms are degraded by autophagy. However, the variant is unable to interact with Keap1. Notably, the variant enhances the E3 activity of cullin-3-based Keap1 ubiquitin ligase and subsequently promotes ubiquitination of Nrf2, leading to its degradation by the 26S proteasome. As a result, the transactivation of Nrf2 is suppressed by the variant. In addition to being unable to competitively inhibit the Keap1-Nrf2 interaction, the variant suppresses the autophagy-dependent degradation of Keap1. Therefore, we propose that splicing of p62 pre-mRNA negatively regulates the Keap1-Nrf2 pathway.

Pre-mRNA splicing is a fundamental process required for expansion of limited genomic information (32). The spliceosome mediates this process through recognition of splicing signals, as well as removal of noncoding intronic sequences, to assemble protein coding sequences into mRNA, which produces timing- and location-specific splicing variants (33). In exon 7 of mouse *p62*, there is a typical 5′ splicing site that is recognized by a component of the spliceosome, i.e., U1 snRNP. If the site is recognized by U1 snRNP, the mRNA coding the variant is generated. If not, full-length p62 mRNA is generated. As shown in Fig. 1F, the variant was induced by oxidative stress. There are two possible explanations for this type of splicing regulation: (i) an oxidative stress-inducible splicing factor(s) may modulate the splicing or (ii) splicing may occur constantly at the same rate irrespective of cellular conditions. In the former case, the variant p62 should be produced at a level above the increased level of full-length p62 under conditions of oxidative stress or impaired autophagy. In the latter case, the elevation of the pre-mRNA level due to stress or autophagy deficiency would cause the same percent increases in the full-length and variant mRNAs. As shown in Fig. 1G and 4C, levels of full-length and variant p62 increased proportionally in response to exposure to arsenite and under autophagy-deficient conditions, supporting the latter hypothesis.

The p62-Keap1-Nrf2 axis is induced under selective autophagy conditions (21). When selective autophagic cargos, such as misfolded proteins and damaged mitochondria, appear in the cytoplasm, they are ubiquitinated (18). Concomitantly, Ser407, located in the UBA domain of p62, is initially phosphorylated by ULK1 kinase (34). This phosphorylation destabilizes the UBA dimer interface (35), and subsequently, casein kinase 2, TANK-binding kinase 1, or ULK1 phosphorylates Ser403 of the UBA domain (34, 36, 37), which increases the binding affinity of p62 for the ubiquitin chain. Consequently, p62 is translocated to the ubiquitinated cargos and then phosphorylated at Ser351 by mTORC1, and Keap1 is sequestered into ubiquitinated cargos, resulting in Nrf2 activation (21, 26). Because Nrf2 regulates the gene expression of *p62* (20), the expression level of *p62* mRNA gradually increases under selective autophagy conditions, leading to a positive-feedback loop in the p62-Keap1-Nrf2 axis. However, because persistent activation of Nrf2 is cytotoxic (11, 30), it is plausible that the pathway is also regulated negatively. In fact, modes of negative regulation of the Keap1-Nrf2 pathway were recently reported (22, 38). When activity of the pathway exceeds a threshold, the splicing variant might become functional, thereby repressing constitutive Nrf2 activation. Indeed, in autophagy-deficient mouse livers, in which Nrf2 is abnormally activated, the variant also prominently accumulated (Fig. 4C). Likewise, the variant form was induced in hepatocytes exposed to arsenite for long periods (Fig. 1G). Furthermore, we confirmed that in primary hepatocytes lacking only variant p62 (i.e., *p62-GFP^KI/KI^* hepatocytes), the expression of Nrf2 target genes was markedly induced by exposure to arsenite and after removal of arsenite persisted at higher levels than those in wild-type hepatocytes (Fig. 6).

Several lines of evidence imply that generation of *de novo* gene products and interaction networks during evolution have created a sophisticated p62-mediated Keap1-Nrf2 pathway (39). p62 is conserved among metazoans but not in plants and fungi, and the KIR is present only in vertebrates, indicating that the p62-Keap1-Nrf2 axis was established in vertebrates. In Caenorhabditis elegans, accumulation of damaged or superfluous mitochondria causes oxidative stress, resulting in activation of SKN-1, a homologue of mammalian Nrf2, to induce the coordinated expression of mitochondrial biogenesis and mitophagy genes to preserve mitochondrial quality (40). Although SKN-1 is committed to the antioxidant response and autophagy, Keap1 is not present in Caenorhabditis elegans. In Drosophila melanogaster, expression of both *Atg8a* and *Ref*(*2*)*P*, which encode homologues of mammalian LC3 and p62, respectively, are regulated by CncC, a homologue of Nrf2; consequently, activation of CncC induces autophagy (41). Remarkably, while Ref(2)P does not have the ability to interact with Keap1, Atg8a interacts directly with Keap1 to modulate its levels through autophagy, thus regulating CncC activity (41). *TRIM21* is conserved in vertebrates, and its expression is regulated by interferons, a family of secreted proteins that exert antiviral and immunomodulatory activities (42). To our surprise, regulation by *p62* pre-mRNA splicing does not seem to be conserved even among mammals and may instead be continuing to evolve. For instance, although not only exon 7 but also the splicing sites necessary for producing the variant are conserved among mice and humans (see Fig. S1A in the supplemental material), reverse transcriptase PCR analysis of HeLa, HEK293T, SH-SY5Y, and U251 cells (Fig. S1B) indicated no KIR splicing variant mRNA in those cell lines (Fig. S1B). Instead, we noticed the presence of a *p62* mRNA encoding a variant, predicted at transcript support level 1, that lacks the N-terminal PB1 domain (http://www.ensembl.org/Homo_sapiens/Info/Index). Further analysis is needed to clarify the physiological role of the human splicing variant.

## MATERIALS AND METHODS

### Cell culture.

Media and reagents for cell culture were purchased from Life Technologies (Grand Island, NY). *p62*-deficient mouse embryonic fibroblasts (MEFs) and control MEFs were prepared as described previously (30). The Hepa-1 and Huh1 cell lines were purchased from the Health Science Research Resources Bank (Osaka, Japan). To generate *p62*-knockout cells, a p62 guide RNA designed by use of the CRISPR Design tool (http://crispr.mit.edu/) was subcloned into pX330-U6-Chimeric_BB-CBh-hSpCas9 (Addgene), a human codon-optimized SpCas9 and chimeric guide RNA expression plasmid. Huh1 and HeLa cells were cotransfected with the pX330 and pEGFP-C1 (Clontech Laboratories, Inc., Mountain View, CA) vectors and cultured for 2 days. Thereafter, the GFP-positive cells were sorted and expanded. Loss of *p62* was confirmed by heteroduplex mobility assay followed by immunoblot analysis with anti-p62 antibody. Mouse p62 and variant adenoviruses were prepared using an adenovirus expression vector kit (TaKaRa Bio, Otsu, Japan).

### Mice.

*Atg7^f/f^*, *Atg7^f/f^*; albumin-*Cre*, and *p62-GFP^KI/KI^* mice in the C57BL/6 genetic background were used in this study (30, 31). Mice were housed in specific-pathogen-free facilities, and the Ethics Review Committee for Animal Experimentation of Niigata University approved the experimental protocol.

### Reverse transcriptase PCR and quantitative real-time PCR.

Using a Transcriptor first-strand cDNA synthesis kit (Roche Applied Science, Indianapolis, IN), cDNA was synthesized from 1 μg of total RNA. LA-*Taq* polymerase (TaKaRa Bio) was used for PCR analysis. The sequences of the primers used were as follows: primer F, GAACATGGAGGGAAGAGAAG; and primer R, TCACAATGGTGGAGGGTGCTTCG. Quantitative PCR was performed using LightCycler 480 Probes master mix (Roche Applied Science) on a LightCycler 480 instrument (Roche Applied Science). Signals from human samples were normalized against the *GAPDH* (glyceraldehyde-3-phosphate dehydrogenase gene) signal. The sequences of the primers used were as follows: *Nqo1* Left, AGCGTTCGGTATTACGATCC; *Nqo1* Right, AGTACAATCAGGGCTCTTCTCG; *Gstm1* Left, CACAAGATCACCCAGAGCAA; *Gstm1* Right, TGGTTCTCCACAATGTCTGC; *Gus* Left, CTCTGGTGGCCTTACCTGA; and *Gus* Right, CTCAGTTGTTGTCACCTTCACC.

### Digital PCR.

cDNA was synthesized as described in “Reverse transcriptase PCR and quantitative real-time PCR.” Absolute quantification was performed using a QuantStudio 3D digital PCR system (Thermo Fisher Scientific) and analyzed with QuantStudio 3D AnalysisSuite cloud software (Thermo Fisher Scientific). The sequences of primers and probes were as follows: *p62* full-length Left, CCCACAGGGCTGAAGGAA; *p62* full-length Right, CATCTGGGAGAGGGACTCAATC; *p62* full-length Probe, CCCACCAGAGGCTGA; *p62* variant Left, CGATGACTGGACACATTTGTCTTC; *p62* variant Right, TCTGGGAGAGGGACTCAATCA; and *p62* full-length Probe, CCATCACAGAGGCTG.

### Immunoblot analysis.

Cells were lysed with ice-cold TNE buffer (50 mM Tris-HCl, pH 7.5, 150 mM NaCl, 1 mM EDTA) containing 1% Nonidet P-40 and protease inhibitors. Samples were separated in 12% Bis-Tris gels in NuPAGE morpholinepropanesulfonic acid (MOPS)-SDS running buffer by use of a NuPAGE system (Invitrogen, Carlsbad, CA) and then transferred to polyvinylidene difluoride (PVDF) membranes. For immunoprecipitation analysis, cells were lysed in 200 μl of TNE buffer, and the lysate was then centrifuged at 10,000 × *g* for 10 min at 4°C to remove debris. In the next step, 800 μl of TNE and 15 μl of FLAG-M2 agarose (Sigma), GFP-agarose (MBL), or anti-Nrf2 antibody (H-300; Santa Cruz Biotechnology, Dallas, TX) were added to the lysate, and the mixture was incubated for 12 h at 4°C with constant rotation. The immunoprecipitates were washed five times with ice-cold TNE buffer. The complex was boiled for 10 min in LDS sample buffer in the presence of 2-mercaptoethanol to elute proteins and then centrifuged at 10,000 × *g* for 10 min at 4°C. The supernatant was subjected to electrophoresis in a NuPAGE gel, followed by immunoblot analysis. Antibodies against p62 (GP62-C; Progen Biotechnik GmbH, Heidelberg, Germany) (diluted 1:1,000), Nqo1 (ab34173; Abcam) (diluted 1:1,000), Ugdh (ab155005; Abcam) (diluted 1:1,000), Nrf2 (H-300; Santa Cruz Biotechnology) (diluted 1:200), LC3B (2775; Cell Signaling Technology, Danvers, MA) (diluted 1:500), Keap1 (10503-2-AP; Proteintech Group, Chicago, IL) (diluted 1:500), actin (MAB1501R; Merck Millipore Headquarters, Billerica, MA) (diluted 1:1,000), and lamin B (M-20; Santa Cruz Biotechnology; diluted 1:200) were purchased from the indicated suppliers. Anti-phosphorylated p62 polyclonal antibody (dilution ratio of 1:500) was raised in rabbits by using the peptide Cys+KEVDP(pS)TGELQSL as an antigen (21). To detect p62-GFP, we used a rabbit polyclonal anti-A170/p62 antibody (30). Blots were then incubated with a horseradish peroxidase-conjugated secondary antibody [goat anti-mouse IgG(H+L) (115-035-166; Jackson ImmunoResearch Laboratories, Inc., West Grove, PA), goat anti-rabbit IgG(H+L) (111-035-144; Jackson ImmunoResearch Laboratories), goat anti-guinea pig IgG(H+L) (106-035-003; Jackson ImmunoResearch Laboratories), or donkey anti-goat IgG(H+L) (705-035-003; Jackson ImmunoResearch Laboratories)] (all diluted 1:10,000) and visualized by chemiluminescence assay.

### Gel filtration chromatography.

Gel filtration chromatography with cell lysates was carried out on a Superose 6 10/300GL column (Äkta Pure 25; GE Healthcare UK Ltd., Little Chalfont, Buckinghamshire, United Kingdom). The column was equilibrated and eluted with TNE buffer containing 0.5% Triton X-100 and protease inhibitors. The collected fractions (1.0 ml) were precipitated with 10% trichloroacetic acid (TCA) and cold acetone. The resultant precipitants were solubilized in 50 μl of LDS-containing sample buffer with 2-mercaptoethanol. Equivalent samples of fractions (fractions 7 to 22) were subjected to immunoblot analysis. Gel filtration standards (conalbumin, 75 kDa; aldolase, 158 kDa; ferritin, 440 kDa; and thyroglobulin, 669 kDa) (GE Healthcare), used as molecular size markers, were loaded under the same experimental conditions.

### Immunocytochemistry.

GFP-tagged full-length or variant p62 was expressed in *p62*-deficient Huh1 cells grown on coverslips. After incubation for 24 h, cells were fixed in 4% paraformaldehyde in phosphate-buffered saline (PBS) for 10 min, permeabilized with 0.1% digitonin in PBS for 5 min, blocked with 0.1% (wt/vol) gelatin (Sigma-Aldrich, St. Louis, MO) in PBS for 30 min, and incubated overnight with primary antibody against Keap1 (10503-2-AP; Proteintech Group). After a washing, cells were incubated with Alexa Fluor-conjugated goat anti-rabbit IgG secondary antibody (A-21245; Life Technologies) for 60 min. Cells were imaged on a confocal laser scanning microscope (FV1000; Olympus, Tokyo, Japan) equipped with a UPlanSApo 100× 1.40-numerical-aperture (NA) oil objective lens. z-projection stack images were acquired with z-steps of 0.5 μm. Image contrast and brightness were adjusted using Photoshop CS4 (Adobe Systems, Inc., San Jose, CA).

### Statistical analysis.

Values, including those displayed in the graphs, represent means ± standard errors of the means (SEM). Statistical analyses were performed using the unpaired *t* test (Welch's *t* test). *P* values of <0.05 denote statistical significance.

## Supplementary Material

Supplemental material
