# Supplementary material for: Negative Regulation of the Keap1-Nrf2 Pathway by a p62/Sqstm1 Splicing Variant
Source: Mol Cell Biol. 2018 Mar 15;38(7):e00642-17. doi: 10.1128/MCB.00642-17 (PMC5854834; doi:10.1128/MCB.00642-17)
Supplement: Supplemental material [file supp_38_7_e00642-17__index.html]

Supplemental material 

# Negative Regulation of the Keap1-Nrf2 Pathway by a p62/Sqstm1 Splicing Variant

## Supplemental material

- Supplemental file 1 -

  Fig. S1 (Alignment of exon 7 and junction region of human and mouse *p62*) and supplemental text

  PDF, 4.3M
