# Supplementary material for: Negative Regulation of the Keap1-Nrf2 Pathway by a p62/Sqstm1 Splicing Variant
Source: Mol Cell Biol. 2018 Mar 15;38(7):e00642-17. doi: 10.1128/MCB.00642-17 (PMC5854834; doi:10.1128/MCB.00642-17)
Supplement: Supplemental material [file MCB.00642-17_zmb999101722s1.pdf]

# Supplementary data

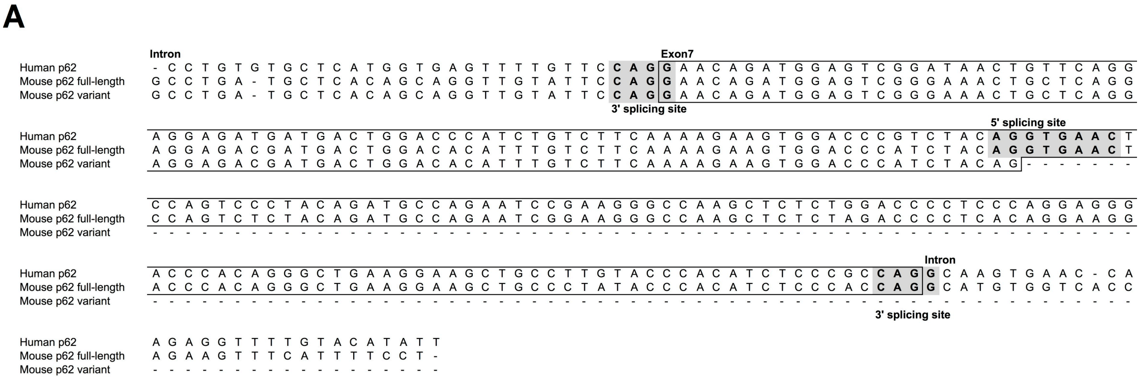


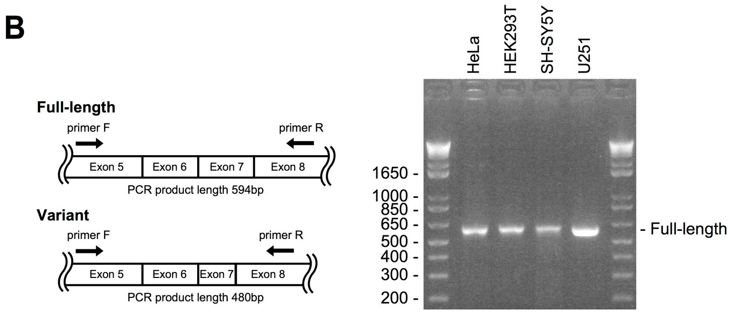


Supplementary Figure 1

(A) Alignment of exon 7 and the junction region of human and mouse *p62*. 5' and 3' splicing sites are indicated.

(B) Reverse-transcriptase (RT)-PCR. Analysis of *p62* exon 7 splicing in HeLa, HEK293T, SH-SY5Y, and U251. Diagrams at left indicate cDNAs encoding full-length and variant p62. The positions of primers used in reverse transcriptase (RT)-PCR of *p62* cDNA and the expected size of the PCR products in the presence or absence of the last half of exon 7 are shown. The right panel shows electrophoresis (2% agarose gel) of RT-PCR products from cDNA of HeLa, HEK293T, SH-SY5Y, and U251.

## Supplementary Materials and Methods

### Reverse-transcriptase PCR

Using the Transcriptor First-Strand cDNA Synthesis Kit (Roche Applied Science, Indianapolis, IN, USA), cDNA was synthesized from 1 μg of total RNA. LA-Taq polymerase (TAKARA BIO.) was used for PCR. The sequences of the primers used were as follows: primer F, GTGGCAGCTGCCCTTAGCCCTC; primer R, TCACAACGGCGGGGGATGCTTTG.
